# Correction to “Glycosylation Deletion of Hemagglutinin Head in the H5 Subtype Avian Influenza Virus Enhances Its Virulence in Mammals by Inducing Endoplasmic Reticulum Stress”

**DOI:** 10.1155/tbed/9847165

**Published:** 2026-08-21

Y. Yin, S. Yu, Y. Sun, et al., “Glycosylation Deletion of Hemagglutinin Head in the H5 Subtype Avian Influenza Virus Enhances Its Virulence in Mammals by Inducing Endoplasmic Reticulum Stress,” *Transboundary and Emerging Diseases*, 2020;67:1492–1506. https://doi.org/10.1111/tbed.13481.

In the article, the authors have identified errors in Figure 6, in which the β‐actin bands shown in Figure 6a were erroneously duplicated from Figure 5. Further investigation by the publisher identified concerns in the western blots presented in Figure 3, which has also been reported on PubPeer [1]. Specifically,

• In Figure 3a, the pIRE1α blot was incorrectly cropped.
• Lanes E and F of the IRE1α blot in Figure 3b and lane F of the β‐actin blot in the right panel of Figure 3d demonstrate undeclared splicing.

These problems resulted from incorrect figure preparation and inappropriate adjustment to the overall brightness and contrast of the image, and the authors have provided the original blots for assessment by the Editorial Board, who agreed that a correction is appropriate. In the corrected Figure 3,

• The pIRE1α blot in Figure 3a and the β‐actin blot in Figure 6a were replaced with the correct images.
• In Figure 3b, both the original images used in the published article and the original images from an independent biological replicate were provided for verification. For clearer presentation and consistency, the corresponding blot from the independent biological replicate was used in the corrected Figure 3b. All panels in the corrected Figure 3 were prepared from the original images; the appearance of some bands may therefore differ from that in the originally published figure (e.g., Bip and IRE1α in Figure 3a). Band intensities were remeasured, and the corresponding quantitative data were corrected accordingly.

These corrections do not affect the interpretation of the results or the conclusions of the article. The corrected Figures 3 and 6 are shown below:

**Figure 3:**
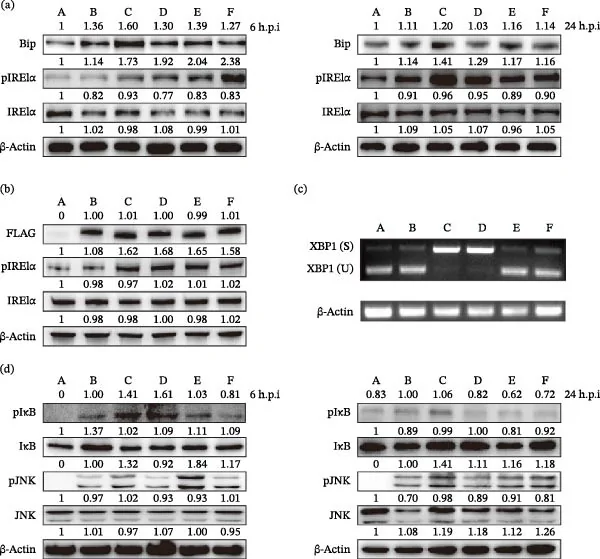
Viruses with de‐glycosylation at head site 158 or 169 induced higher levels of ER stress. AIV infection (a) or HA transfection (24 h post‐transfection, b) activated IRE1α pathway and its downstream XBP1 (c), JNK (d) and NF‐κB (d) pathways. Lysates of A549 cells infected with tested viruses at 1 MOI. Samples collected at 6 and 24 h.p.i. were incubated with BiP, IRE1α, phosphorylated IRE1α, JNK, phosphorylated JNK and IκB, phosphorylated IκB antibodies. Lysates of A549 cells transfected with different glycosylation HA plasmids were incubated with IRE1α, phosphorylated IRE1α and FLAG antibodies at 4°C overnight. β‐Actin served as an internal standard. Bands were visualized by chemiluminescence imaging analysis system after incubation with peroxidase‐conjugated secondary antibodies. Total RNA was extracted from A549 cells infected with tested viruses at 1 MOI at 6 and 24 h.p.i. for transcription, amplification and digestion with PstI. Spliced and unspliced XBP1 were detected by UV illumination. Each experiment was repeated three independent times, and one representative result was shown (A: Mock; B: rS‐144−/158+/169+(WT); C: rS‐144−/158−/169+; D: rS‐144+/158−/169+; E: rS‐144−/158+/169−; F: rS‐144+/158+/169−).

**Figure 6:**
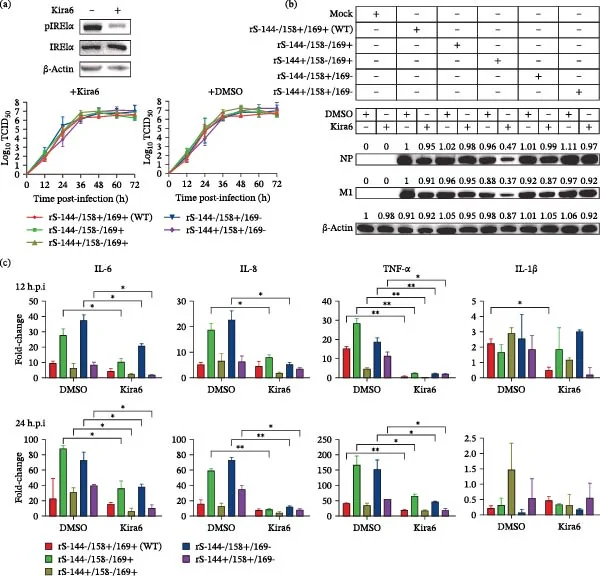
Kira6 functions on ER stress suppression and inflammation alleviation in cells. Effects of Kira6 treatment on the virus growth (a), viral protein expressions (b) and transcriptional expressions of target genes in ER stress response‐related inflammation (c). A549 cells were treated with 1 μM Kira6 post‐infection with respective tested virus. Cell lysates were incubated with IRE1α and phosphorylated IRE1α antibodies individually for interference confirmation and incubated with the M1 and NP proteins for viral protein expression detection. β‐Actin served as an internal standard. Cell supernatants were collected for determination of the TCID_50_ titres at the indicated time points. Cell lysates were subjected to qRT‐PCR analyses of the mRNA expression of the target genes ( ^∗^ *p* < 0.05,  ^∗∗^ *p* < 0.01).

We apologize for these errors.
